# Effect of long-term exercise on circulating ghrelin in overweight and obese individuals: a systematic review and meta-analysis

**DOI:** 10.3389/fnut.2025.1518143

**Published:** 2025-01-23

**Authors:** Xianyang Xin, Hai Wang, Yongqing Guo, Jun Xie

**Affiliations:** Capital University of Physical Education and Sports, Beijing, China

**Keywords:** ghrelin, exercise, overweight and obese, long-term exercise, appetite, meta-analysis

## Abstract

**Objective:**

Ghrelin, also known as the “hunger hormone,” is a pivotal hormone in controlling appetite, and it is the only known gastrointestinal hormone that promotes food intake, contributing to the regulation of energy balance and body weight. However, studies on the long-term effects of exercise on ghrelin levels in obese populations have shown conflicting results. This study aims to summarize RCT experiments exploring changes in ghrelin levels following long-term exercise in obese or overweight individuals through meta-analysis.

**Methods:**

This study employed meta-analytical techniques, searching databases such as PubMed, Web of Science, and EMBASE, to gather research on exercise and ghrelin. The quality of the studies was assessed according to the Cochrane Handbook standards, and data analysis for ghrelin, BMI, and weight was performed using RevMan 5.4 and Stata 16.0 software. A total of 13 interventions involving 944 participants were included to systematically investigate the regulatory effects of exercise on ghrelin levels in obese and overweight individuals. Meta-analytical results were calculated using standardized mean differences (SMDs).

**Results:**

Exercise interventions significantly increased ghrelin levels (SMD =1.16, 95% CI = 0.52 to 1.80, *p* < 0.0001), with high inter-study heterogeneity (*I*^2^ = 90%). Subgroup analysis suggested that RT and AE + RT were more effective than AE. For BMI, exercise led to a significant reduction (SMD = −0.43, 95% CI = −0.69 to −0.16, *p* = 0.002), with low heterogeneity (*I*^2^ = 21%). Similarly, exercise significantly reduced weight (SMD = −0.54, 95% CI = −0.98 to −0.11, *p* = 0.01), though with high heterogeneity (*I*^2^ = 75%). These results suggest exercise effectively improves ghrelin levels, BMI, and weight.

**Conclusion:**

Prolonged exercise interventions demonstrated a statistically significant effect on ghrelin levels. This indicates that exercise interventions can elevate ghrelin levels, which may be associated with reductions in BMI and weight.

**Systematic review registration:**

https://www.crd.york.ac.uk/prospero/, CRD42024588259.

## 1 Introduction

Obesity is recognized as a chronic metabolic disease, with a prevalence that continues to rise globally (1). It increases the risk of cancers, metabolic diseases, and musculoskeletal disorders (2). The latest forecasts by the World Health Organization predict that by 2030, approximately 60% of the global population will be obese or overweight, underscoring the need for innovative treatment approaches (3, 4). Obesity results from an imbalance in the energy balance system, where energy intake from food exceeds energy expenditure (5). The regulation of energy balance and body weight is a complex process influenced by factors such as physical activity, dietary intake, neural, endocrine, and metabolic influences.

Exercise increases energy expenditure (6). Although endurance exercise is widely recommended for weight control (7, 8), the outcomes have been disappointing (9). Reports suggest that weight changes in obese and overweight subjects are below the predicted values based on stipulated energy expenditure (10), partly due to compensatory increases in energy intake (11, 12). Exercise, while beneficial for increasing energy expenditure, has also been shown to influence appetite and overall energy consumption (13, 14). Some researchers have demonstrated that prolonged exercise training may increase the drive to eat when fasting (15–18). Thus, appetite is one of the most influential factors in energy balance, with its regulation playing a crucial role in controlling energy equilibrium (19). Ghrelin, secreted by the gastrointestinal system during hunger, is the only known gut hormone that stimulates appetite (orexigenic properties) and is involved in regulating energy and body weight balance (10). The compensatory metabolic changes during weight loss, including increases in ghrelin, are considered reasons for weight regain and the difficulty in maintaining long-term weight loss effects. Meanwhile, many studies have shown that fasting plasma ghrelin levels are significantly lower in obese subjects than non-obese ones (20–22). Ghrelin’s circulation correlates positively with body weight (23) (higher levels with lower weight) and typically responds compensatorily to weight changes (increases when weight decreases; decreases when weight increases) (13, 14). Long-term exercise in obese populations not only directly increases energy expenditure and reduces weight but also impacts appetite and energy intake, potentially inducing increases in appetite and energy intake, leading to weight changes in obese and overweight subjects that are lower than predicted, even causing weight regain after dieting (24–27). Therefore, exploring the mechanisms of how physical exercise affects eating behaviors in obese populations is crucial for weight loss and reduction.

While intuitively exercise may appear to increase appetite and energy intake, the connection between exercise and appetite is intricate. There is still significant controversy regarding the long-term effects of exercise on ghrelin levels in different populations. Various studies have observed that long-term exercise can lead to an increase (28, 29), a decrease (30–33), or no change (34, 35) in ghrelin levels. The research results are similarly contradictory among obese populations. For instance, a study conducted a 4-week high-intensity interval and sustained exercise intervention on 15 men, showed no significant differences in ghrelin levels pre- and post-exercise (36), with levels tending to decrease. Similarly, a study observed no significant changes in ghrelin levels after an 8-week aerobic exercise intervention (37). A study had participants engage in endurance training, increasing their energy expenditure by 300 kcal/day (MOD group) or 600 kcal/day (HIGH group); both groups observed an increase in ghrelin post-exercise intervention (MOD: *p* = 0.036; HIGH: *p* = 0.027) (38). Current reviews have failed to pinpoint specific populations or types of exercise that are definitively related to changes in circulating ghrelin (39). Previous narrative reviews have suggested that chronic exercise does not affect ghrelin concentrations and is unrelated to weight loss (40, 41). One review argued that exercise training elevates sensations of fasting hunger and post-meal satiety while also enhancing the relationship between energy expenditure and energy intake, allowing energy expenditure to adjust in response to food consumption (25). Long-term training tends to increase total and acylated ghrelin secretion in overweight/obese individuals (21, 42, 43), a form of long-term exercise that can more effectively reduce body weight/fat, especially in obese individuals (43, 44). Many studies also suggest that weight loss is the primary driver of changes in ghrelin levels (21, 29, 45, 46). However, some studies show that while weight may decrease, total circulating ghrelin levels do not change (29, 47–50) or decrease (31, 51). A systematic review has found that both normal-weight and obese patients experience increases in ghrelin levels due to weight loss and suggests that altering this weight-regulating hormone is a compensatory strategy to maintain weight stability (52), but no meta-analysis has yet confirmed this perspective. A meta-analysis targeting diabetic patients indicated that physical exercise had no significant change in ghrelin levels (53). A recent meta-analysis also stated that exercise training programs for overweight and obese patients did not affect total ghrelin levels, and due to the inclusion of other appetite hormone data for BMI and weight, it did not definitively explore the relationship between weight or body mass index changes and ghrelin (54).

Thus, these experimental results and narrative studies are highly controversial. The systematic review and meta-analysis aim to quantify the effects of long-term exercise training (duration ≥4 weeks) on ghrelin in overweight and obese adults and include only studies on the ghrelin hormone with BMI and weight data to analyze the relationships among changes in ghrelin, BMI, and weight, thereby filling an evidence-based gap.

## 2 Methods

This study was conducted following the Preferred Reporting Items for Systematic Reviews and Meta-Analyses (PRISMA) guidelines (55). The review is registered with PROSPERO under the ID 2024-CRD42024588259.^1^

### 2.1 Literature sources and retrieval

The literature search encompassed several databases: PubMed, Web of Science (utilizing both free-text and MeSH terms), China National Knowledge Infrastructure (CNKI), Wanfang Database, MEDLINE, EMBASE, and Cochrane Library. The search took place from 2020 to October 2024. To avoid missing eligible literature, we examined the list of references for systematic reviews published in the last 3 years (39, 52, 54). This search spanned the last 20 years, using keywords such as “ghrelin,” “appetite,” “chronic exercise,” “exercise,” “obesity,” “overweight,” “aerobic exercise,” “resistance exercise,” and “combination exercise” to ensure thorough coverage.

### 2.2 Study selection

During the initial screening, all retrieved records are imported into reference management software (EndNote, X9) to eliminate duplicate records. The two researchers (XY and HW) then independently screened the titles and abstracts to identify all potentially relevant studies and supplemented any missing data by contacting the authors. Studies that met the inclusion criteria were independently identified and evaluated by the same two investigators (XY and HW). Differences are resolved through discussion, with a third expert (JX) consulted if necessary.

Study inclusion criteria were as follows: (1) peer-reviewed; (2) English language; (3) human participants; (4) the study design had to be a randomized controlled trial and had to have a blank control group; (5) participants had to be obese or overweight; (6) the intervention could include any type of exercise training; (7) the control group does not perform the exercise intervention or minor stretching exercises; (8) the study must report ghrelin; and (9) the exercise interventions must have a duration of more than 4 weeks, as evidence suggests that this duration is sufficient to achieve measurable changes in body weight and body fat (56, 57). Study exclusion criteria were as follows: (1) if they had a medical condition (diabetes, hypertension, arthritis, etc.) or a special group (pregnant women, post-surgery); (2) if multiple articles from the same study reported the same or overlapping results, we included only the most recently published article; and (3) if exercise interventions were combined with diet or a hypoxic environment.

### 2.3 Data extraction

Data extraction was conducted independently by two researchers (XY and YQ) using a self-designed statistical form based on the Cochrane Handbook. The form included study characteristics (first author’s name, year of publication, title, study design, and setting), participant characteristics (sample size, mean age, and sex ratio), interventions (type, frequency, intensity, duration per session, time of intervention, and comparator information), and outcomes (relevant statistics for the intervention endpoints used to estimate effect sizes, such as mean, standard deviation, and corresponding measurement instruments). When relevant statistics were incompletely reported, we estimated the mean and standard deviation based on sample size, median, extreme variance, and *p*-value according to the Cochrane Handbook. When inconsistent units of measurement were encountered, we also performed unit conversions.

### 2.4 Quality assessment

In meta-analysis, using robust quality assessment methods is crucial for determining whether the study designs and procedures in the included literature adhere to scientific standards. This research adopted the Cochrane criteria for evaluation, focusing on the risks associated with random sequence generation, allocation concealment, blinding of participants and personnel, blinding of outcome assessment, incomplete outcome data, selective reporting, and other potential biases. Each element was thoroughly assessed and classified as high risk, low risk, or unclear. This rigorous risk assessment process ensures the credibility of the study and upholds the scientific integrity of the findings.

### 2.5 Statistical analysis

Data analysis was performed using Review Manager 5.4 (58) and Stata 16.0 software. To account for variations across different experiments, the effect size was expressed as the weighted mean difference (WMD) with a 95% confidence interval. Heterogeneity was assessed using the consistency coefficients P and I^2^. Due to statistical heterogeneity among the study groups, but not clinical heterogeneity, a random-effects model was applied. Sensitivity analysis was also conducted to mitigate the impact of bias from individual studies on the overall effect.

## 3 Results

### 3.1 Literature search results

**Figure 1:**
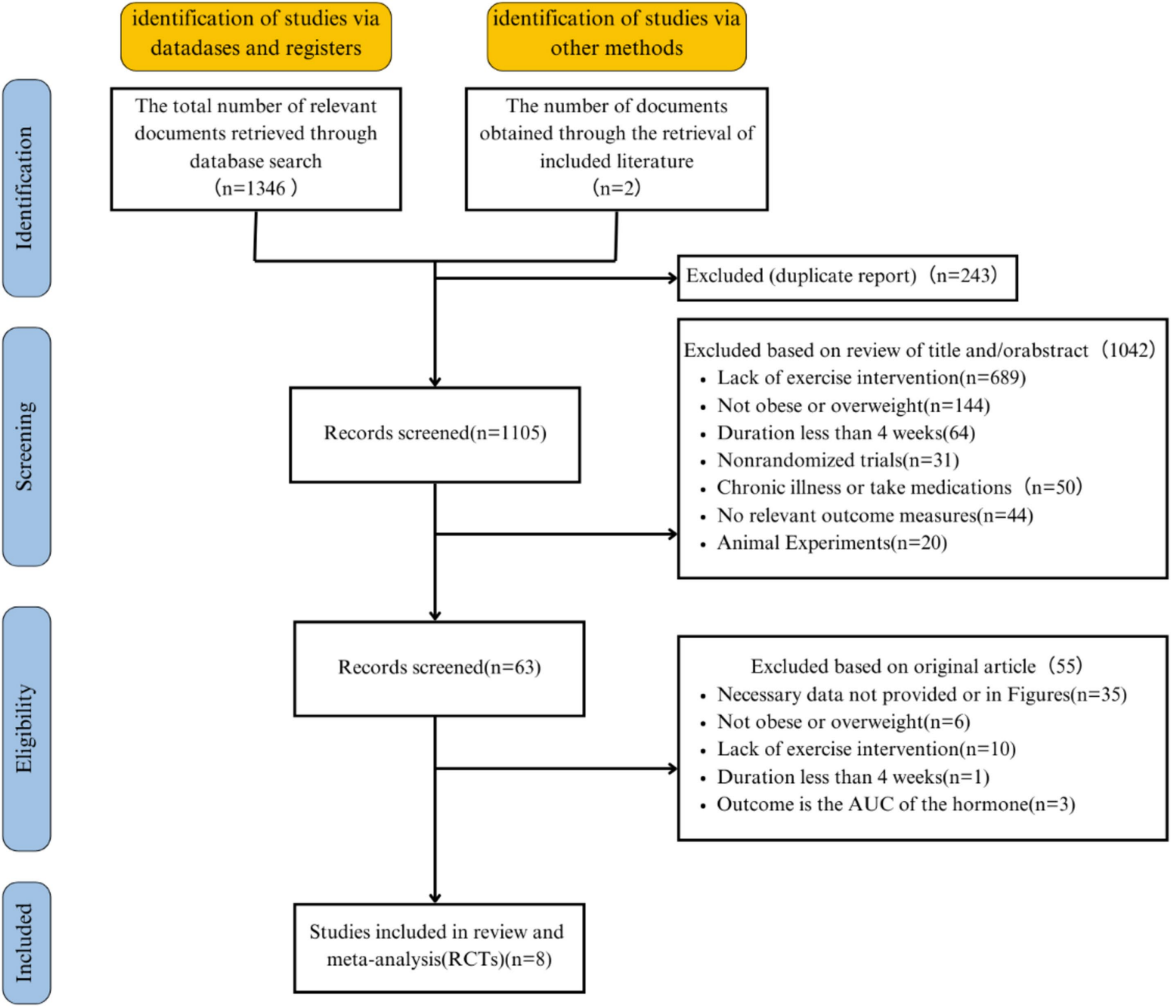
Flowchart of the studies selection process.

A total of 1,346 articles were obtained from the database, along with two additional articles identified through reference searches, resulting in an initial retrieval of 1,348 articles. A total of 243 duplicate studies were removed, leaving 1,105 studies for initial title and abstract screening. Sixty-three studies underwent full-text screening, and after the screening process, 8 articles with 13 interventions were ultimately selected for inclusion (21, 22, 37, 38, 43, 59–61). Figure 1 illustrates the literature selection process. The 10 eligible studies are descriptively summarized in Table 1.

**Table 1:** Overview of basic characteristics of included literature in meta-analysis.

Author (publication year)	Country	Research type	Age		BMI		Weight		Sex		Sample size	
			Exercise	Control	Exercise	Control	Exercise	Control	Exercise	Control	Exercise	Control
Ahmadi et al. (37)	Iran	RCT	69.13 ± 3.6	69.20 ± 4.3	27.57 ± 1.5	27.07 ± 1.1	NR	NR	Male	Male	15	15
Rosenkilde et al. (38)(1)	Denmark	RCT	30 ± 7	31 ± 6	28.6 ± 1.8	28.0 ± 2.3	93.2 ± 8.1	92.8 ± 8.5	Male	Male	21	18
Rosenkilde et al. (38)(2)	Denmark	RCT	28 ± 5	31 ± 6	27.6 ± 1.4	28.0 ± 2.3	91.3 ± 7.2	92.8 ± 8.5	Male	Male	22	18
Maleki and Safarzade (62),	Iran	RCT	30–45	30–45	30 ± 2.8	31.7 ± 4.1	77.31 ± 6.1	76.7 ± 12.3	Female	Female	8	8
Kang et al. (21)	Korea	RCT	50.15 ± 3.82	49.84 ± 2.96	31.8 ± 3.2	30.4 ± 2.3	82.7 ± 10.4	77.2 ± 9.6	Female	Female	13	13
Mason et al. (22)	United States	RCT	57.74 ± 4.4	58.1 ± 5	30.7 ± 3.9	30.7 ± 3.7	84.2 ± 12.5	83.7 ± 12.3	Female	Female	105	80
Foster-Schubert et al. (43)	United States	RCT	60.7 ± 6.7	60.6 ± 6.8	30.4 ± 4.1	30.5 ± 3.7	81.4 ± 14.1	81.7 ± 12.1	Female	Female	83	85
Ataeinosrat et al. (59)(1)	Iran	RCT	27.5 ± 9.4	27.5 ± 9.4	32.4 ± 1.4	32.9 ± 1.4	92.9 ± 2.8	93.8 ± 2.0	Male	Male	11	11
Ataeinosrat et al. (59)(2)	Iran	RCT	27.5 ± 9.4	27.5 ± 9.4	33 ± 1.2	32.9 ± 1.4	92.4 ± 1.9	93.8 ± 2.0	Male	Male	11	11
Ataeinosrat et al. (59)(3)	Iran	RCT	27.5 ± 9.4	27.5 ± 9.4	33.1 ± 0.7	32.9 ± 1.4	93.7 ± 1.9	93.8 ± 2.0	Male	Male	11	11
Mager et al. (61)(1)	United States	RCT	60 ± 7	60 ± 7	33.5 + 3.1	32.4 + 2.5	92.7 + 11.2	87.9 + 8.3	7/7(M/F)	8/10(M/F)	14	18
Mager et al. (61)(2)	United States	RCT	60 ± 7	60 ± 7	32.8 ± 2.2	32.4 + 2.5	97.7 ± 12.9	87.9 + 8.3	10/5(M/F)	8/10(M/F)	15	18
Kim et al. (60)	Korea	RCT	2–18	2–18	25.7 ± 5.04	24.3 ± 0.42	58.3 ± 14.9	59.8 ± 1.5	Boys	Boys	8	9

Age in years, BMI in Kg/m^2^, weight: Kg, VO_2max_, maximal rate of oxygen consumption. BMI, body mass index; NR, not reported.

### 3.2 Quality assessment results

**Figure 2:**
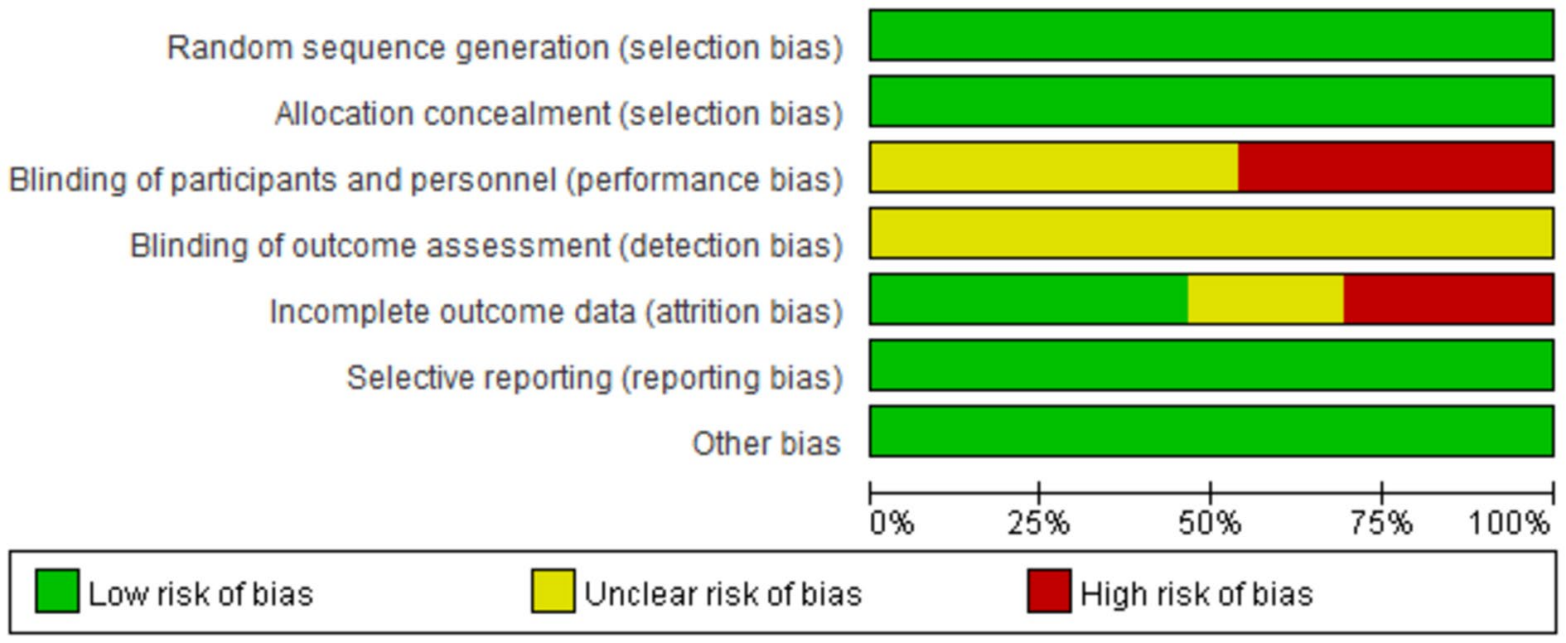
Proportional representation of risk of bias assessment in included trials.

**Figure 3:**
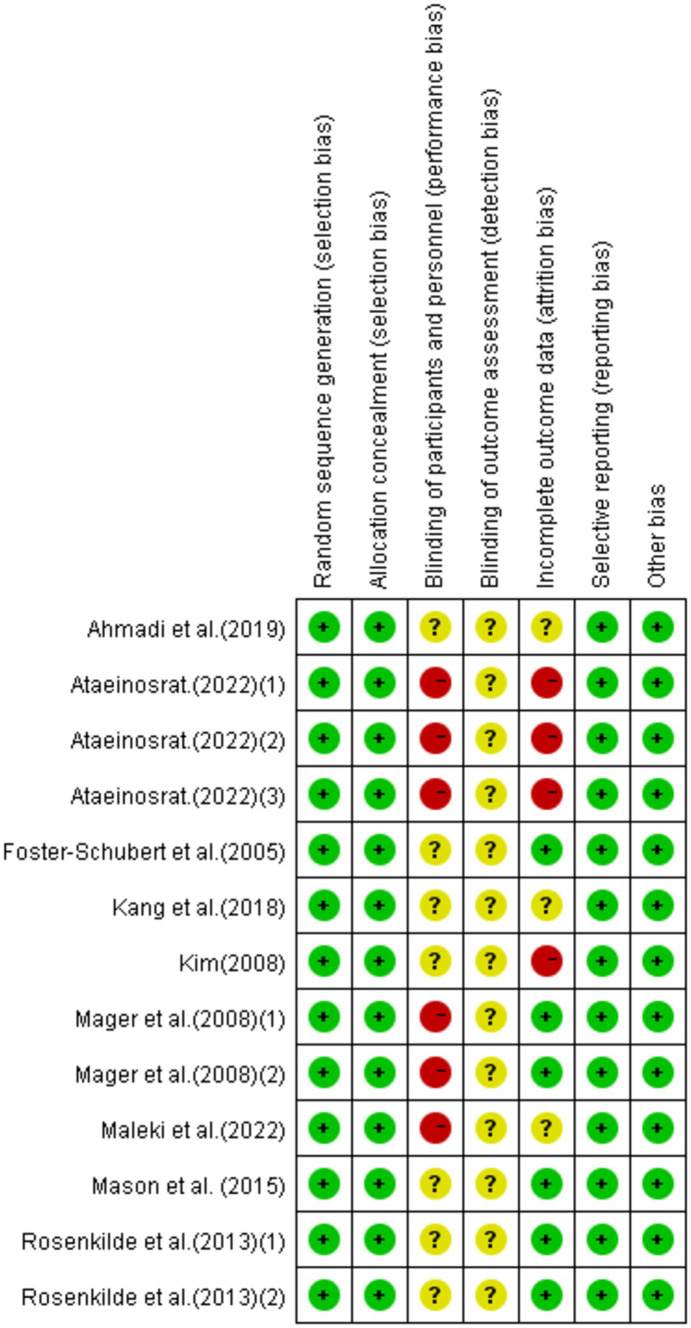
Schematic representation of risk of bias assessment for included trials.

Eight research articles with 13 interventions were included in this study. Each intervention was assessed based on Cochrane standards as illustrated in Figures 2, 3. All 13 interventions were conducted using a randomized control design” to ensure proper grammar and to clearly convey that all 13 interventions utilized a randomized control design (21, 22, 37, 38, 43, 59–62). The assessment showed that all included articles mentioned the generation of random sequences, categorizing them as low risk. All studies performed concealed allocation of random sequences. None of the articles went into detail about double blindness between implementers and participants, and six interventions explicitly mentioned an unblinded setting (59, 61, 62). In studies where the intervention included only two different exercises or a single exercise, blinding was challenging to implement, making it difficult to conceal the purpose of the study. Five studies reported participant withdrawals (22, 38, 43, 59, 60). Three studies had withdrawals unrelated to the experimental intervention, with a withdrawal rate of less than 20% (22, 38, 43), so they were considered low-risk studies. Two articles had more than 20% interruptions and were classified as high risk (59, 60). All articles were free from selective reporting and other biases and thus were categorized as low risk.

Note:

1. Generation of random sequences (selection bias);
2. Allocation concealment (selection bias);
3. Double-blinding of implementers and participants (implementation bias);
4. Blinding in outcome assessment (detection bias);
5. Incomplete outcome data (attrition bias);
6. Selective reporting (reporting bias);
7. Other biases (excluding other important biases from the biases mentioned above).

### 3.3 Basic characteristics of the included studies

This meta-analysis included 8 articles and 13 interventions, as detailed in Tables 1, 2. All of the articles were published in English-language journals, covering the period from 2005 to 2022. The studies included a total of 643 participants ranging in age from 2 to 72.81 years, with only one study <18 years of age. The participants mainly exhibited high levels of body fat, indicating characteristics of obesity and overweight. For the children in the study (60), their weight had to be at or above the 85th percentile of the body mass index (BMI) value in the growth chart for Korean children aged 2–18 years; the remaining articles had a BMI of 26.1 to 34.3 kg.m^−2^.

**Table 2:** Overview of research methodology design and outcome measures in included literature for meta-analysis.

Author (publication year)	Mode	Frequency	Intensity	Duration	Sample source	Test method	Test time	Outcome	
								Exercise	Control
Ahmadi et al. (37)	AE	4/7, 50–60 min	Intensity 60% ~ 85% of maximum heart rate	8 weeks	Plasma	Measured using an ELISA	24 h after the last training session	↑Ghrelin ↓BMI	↔Ghrelin ↔BMI
Rosenkilde et al. (38)(1)	AE	7/7	Increase exercise exertion by 300 kcal per day on top of individual endurance exercise prescription	7 weeks	Plasma	using Radioimmunoassay with commercially available kits	measured after an overnight fast on a separate day and have been described elsewhere	↔Ghrelin ↓BMI ↓Weight	↔Ghrelin ↔BMI ↔Weight
Rosenkilde et al. (38)(2)	AE	7/7	Increase exercise exertion by 600 kcal per day on top of individual endurance exercise prescription	7 weeks	Plasma	Using radioimmunoassay with commercially available kits	measured after an overnight fast on a separate day and have been described elsewhere	↔Ghrelin ↓BMI ↓Weight	↔Ghrelin ↔BMI ↔Weight
Maleki and Safarzade (62)	RE	3/7	50% 1-RM gradually increased to 80–85% 1-RM	8 weeks	Serum	Assessed by the enzyme-linked immunosorbent assay	72 h after the last exercise	↔Ghrelin ↔BMI ↔Weight	↔Ghrelin ↑BMI ↑Weight
Kang et al. (21)	AE + RE	5/7,50 min	set to 12–14, based on the rating of perceived exertion	12 weeks	Serum	Using a chemistry analyzer	confirming the fasting state for 8 h	↑Ghrelin ↓BMI ↓Weight	↓Ghrelin ↔BMI ↔Weight
Mason et al. (22)(1)	AE	5/7,45 min	60 70% to 70 85% HR maximum duration	12 months	Serum	Immune-reactive serum ghrelin by radioimmunoassay using the Millipore total human ghrelin assay	requested not to exercise for the preceding 24 h	↔Ghrelin ↓BMI ↓Weight	↔Ghrelin
Foster-Schubert et al. (43)	AE	5/7	The training program started at 40% of the maximal heart rate for 16 min/session and gradually increased to 60–75% of the maximal heart rate for 45 min/session by week 8, where it was maintained for the duration of the study	12 months	Plasma	Uses a polyclonal antibody raised against full-length acylated human ghrelin and 125I-labeled ghrelin as a tracer	Collected morning blood samples before breakfast after a 12-h overnight fast	↑Ghrelin ↓Weight	↔Ghrelin ↔Weight
Ataeinosrat et al. (59)(1)	RE	3/7,70 min	At an intensity of 50% of 1-RM, rest for 30 s each exercise	12 weeks	Plasma	Measured using an ELISA	using standard procedures following a 12-h overnight fast	↑Ghrelin ↔BMI ↔Weight	↔Ghrelin ↔BMI ↔Weight
Ataeinosrat et al. (59)(2)	RE	3/7,70 min	at an intensity of 50% of 1-RM, rest for 15 s each exercise	12 weeks	Plasma	Measured using an ELISA	using standard procedures following a 12-h overnight fast	↑Ghrelin ↔BMI ↓Weight	↔Ghrelin ↔BMI ↔Weight
Ataeinosrat et al. (59)(3)	RE	3/7,70 min	at an intensity of 50% of 1-RM, active rest with 25% of 1-RM, and 14 repetitions	12 weeks	Plasma	Measured using an ELISA	using standard procedures following a 12-h overnight fast	↑Ghrelin↓BMI ↓Weight	↔Ghrelin ↔BMI ↔Weight
Mager et al. (61)(1)	AE	3–4/7	at 60–70% of 1-RM	33 weeks	Plasma	Measured by RIA using a commercially available kit	overnight fasting plasma levels of ghrelin	↔Ghrelin ↔BMI ↔Weight	↓Ghrelin ↔BMI ↔Weight
Mager et al. (61)(2)	RE	>4/7,30 min	at 55–65% of the maximum level	33 weeks	Plasma	Measured by RIA using a commercially available kit	overnight fasting plasma levels of ghrelin	↔Ghrelin ↔BMI ↔Weight	↓Ghrelin ↔BMI ↔Weight
Kim et al. (60)	AE + RE	4/7	as aerobic exercise, was performed two times a week (Monday and Thursday) at 55–64% and 65–75% of maximum heart rate, resistance exercise for 50 min two times a week	12 weeks	Plasma	Measured with a commercial RIA kit using 125I-labeled bioactive ghrelin as a tracer molecule and a polyclonal rabbit antibody against full-length octanoylated human ghrelin	after a 12-h fast	↑Ghrelin ↔BMI ↔Weight	↔Ghrelin ↔BMI ↔Weight

AE, aerobic exercise; RE, resistance exercise; AE + RE, aerobic exercise + resistance exercise; VO_2max_, maximal rate of oxygen consumption; BMI, body mass index; NR, not reported.

All included studies had an intervention duration of more than 4 weeks, and intervention duration ranged from 7 to 48 weeks. The meta-analysis included three forms of exercise in the studies, aerobic exercise, resistance exercise, and a combination of aerobic and resistance exercise. The meta-analysis primarily investigated the effects of chronic exercise on ghrelin, BMI, and weight, incorporating 13, 11, and 11 studies, respectively. None of the interventions involved diet or hypoxia.

### 3.4 Effects of the interventions on ghrelin, BMI, and weight

#### 3.4.1 Ghrelin

**Figure 4:**
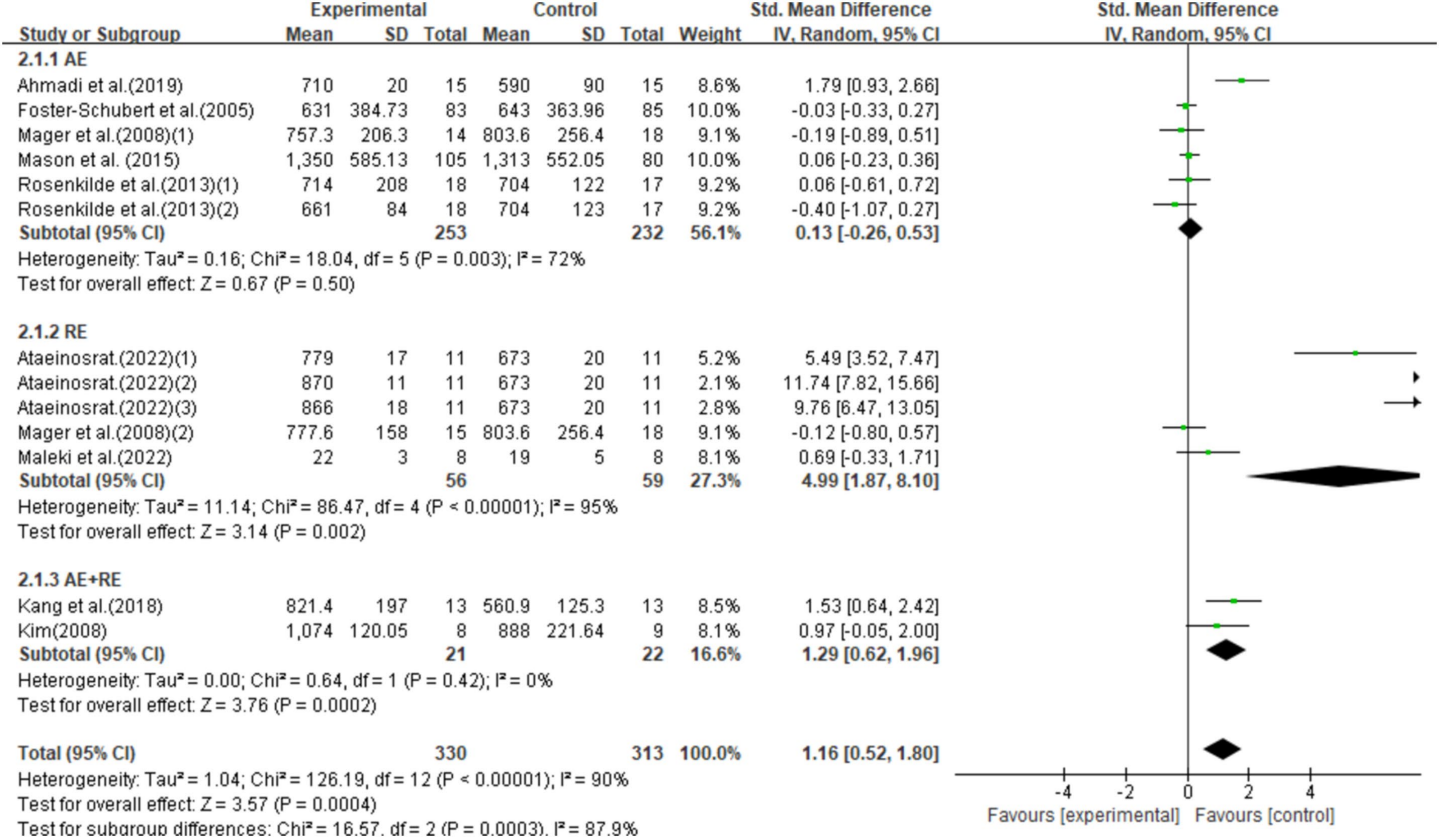
Forest plot of the effect of exercise training interventions on ghrelin compared with non-exercise controls.

This study included 13 studies with 643 participants, finding that exercise interventions significantly increased ghrelin levels (SMD = 1.16, 95% CI = 0.52–1.80, *p* < 0.0001) but with high heterogeneity (I^2^ = 90%). Subgroup analysis showed that AE had no significant effect on ghrelin (SMD = 0.13, 95% CI = −0.26–0.53, *p* = 0.50), while RE and AE + RE significantly increased ghrelin levels [SMD = 4.09, 95% CI = 1.87–8.10, *p* = 0.002] and [SMD = 1.29, 95% CI = 0.62–1.96, *p* = 0.0002], respectively. Significant differences in effects among exercise modalities suggest the need for further standardized research to validate these findings (Figure 4).

#### 3.4.2 BMI

**Figure 5:**
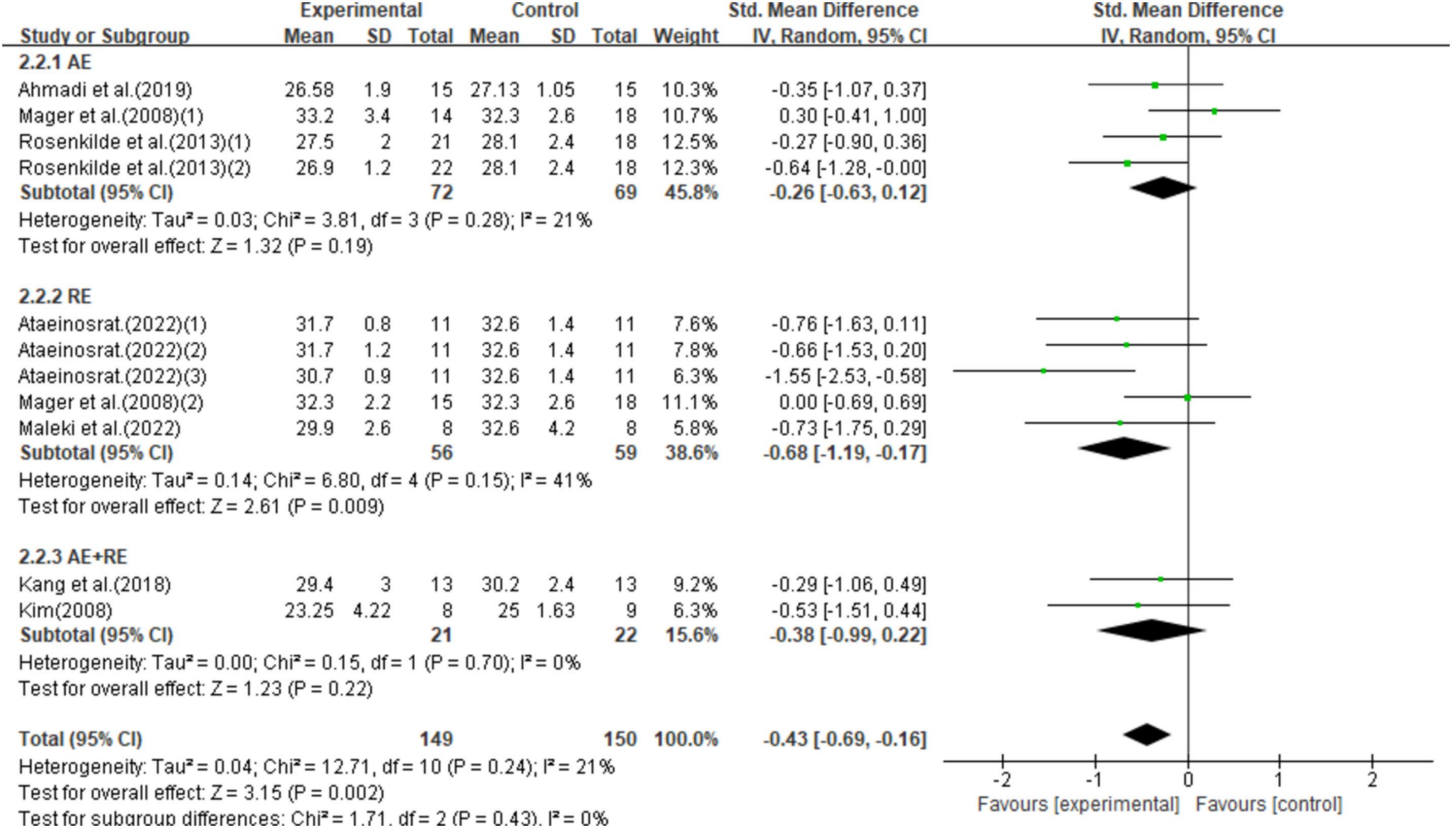
Forest plot of the effect of exercise training interventions on BMI compared with non-exercise controls.

This study included 11 studies with 299 participants, showing that exercise interventions significantly reduced BMI (SMD = −0.43, 95% CI = −0.69 to −0.16, *p* = 0.002) with low heterogeneity (*I*^2^ = 21%). Subgroup analysis found that AE had no significant effect on BMI (SMD = −0.26, 95% CI = −0.63–0.12, *p* = 0.19), while RE significantly reduced BMI (SMD = −0.68, 95% CI = −1.19–-0.17, *p* = 0.009), and AE + RE showed no significant effect (SMD = −0.38, 95% CI = −0.99–0.22, *p* = 0.22; Figure 5).

#### 3.4.3 Weight

**Figure 6:**
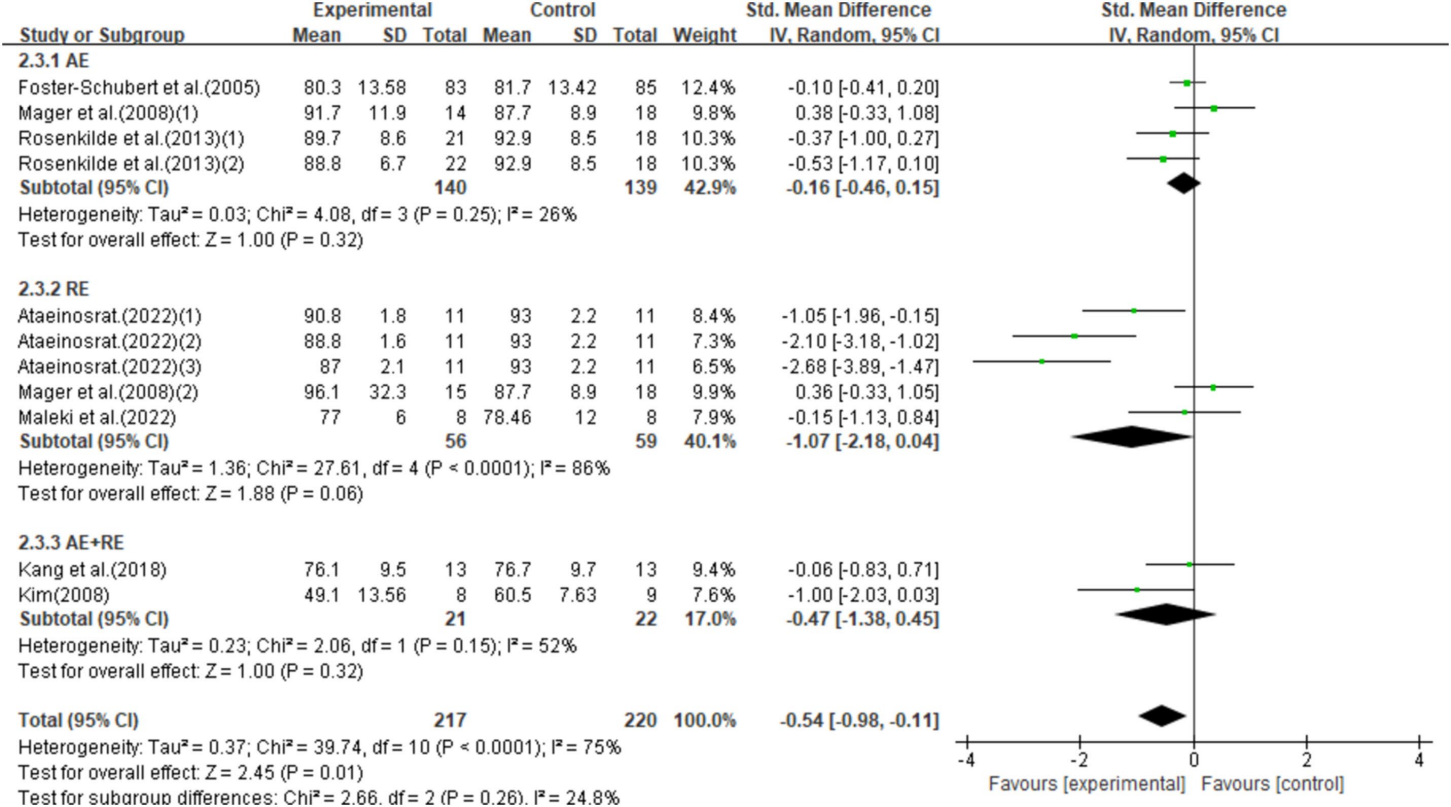
Forest plot of the effect of exercise training interventions on weight compared with non-exercise controls.

This study included 11 studies with 437 participants and found that exercise interventions significantly reduced weight (SMD = −0.54, 95% CI = −0.98–-0.11, *p* = 0.01), but with high heterogeneity (*I*^2^ = 75%). Subgroup analysis showed that aerobic exercise (AE) had no significant effect (SMD = −0.16, 95% CI = −0.46–0.15, *p* = 0.32), while resistance exercise (RE) showed a notable trend toward weight reduction (SMD = −1.07, 95% CI = −2.18–0.04, *p* = 0.06). Combined aerobic and resistance exercise (AE + RE) did not show a significant effect (SMD = −0.47, 95% CI = −1.38–0.45, *p* = 0.31; Figure 6).

### 3.5 Publication bias and sensitivity analysis

**Figure 7:**
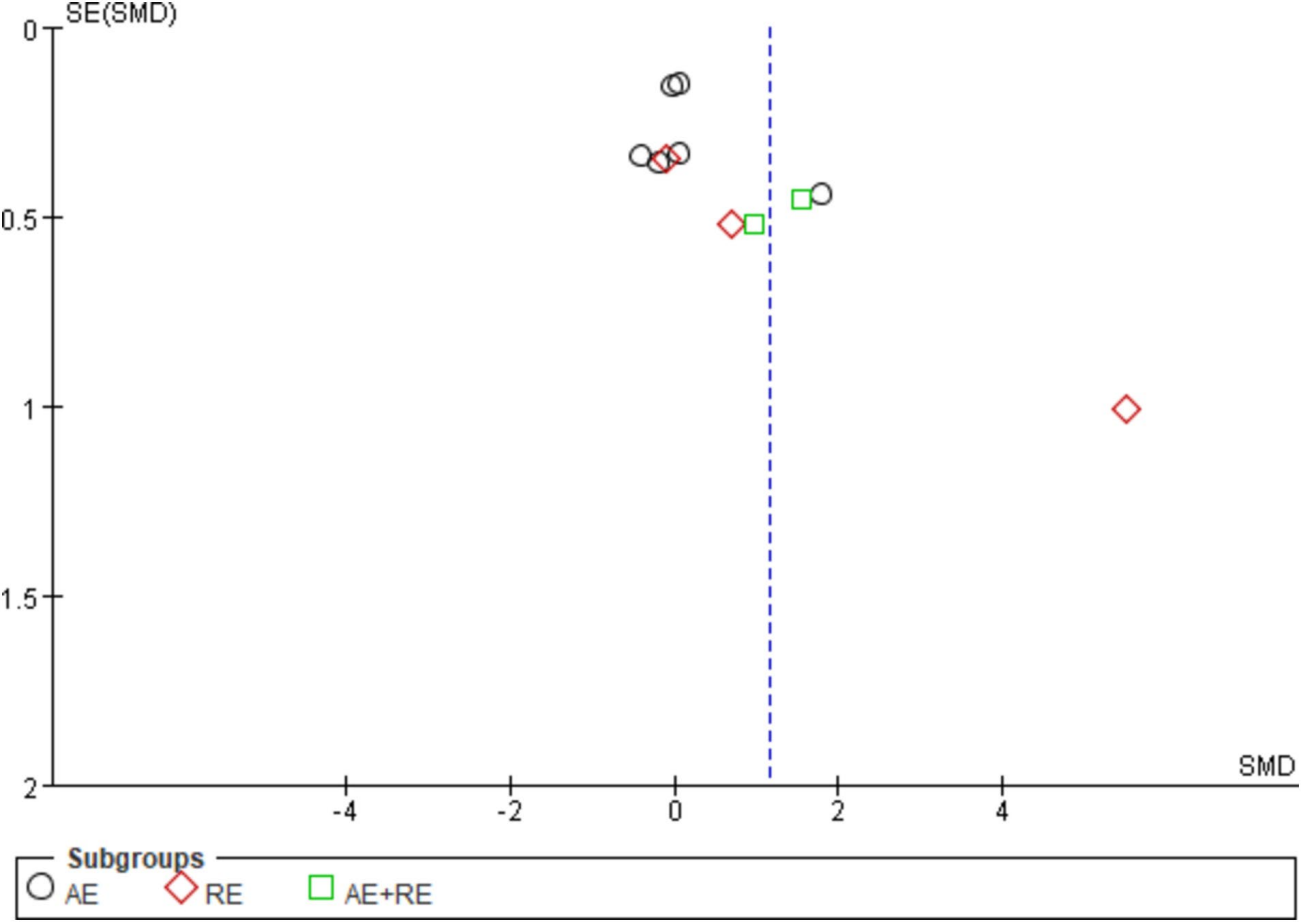
Funnel plot illustrates the effect of exercise on ghrelin in obese and overweight individuals.

**Figure 8:**
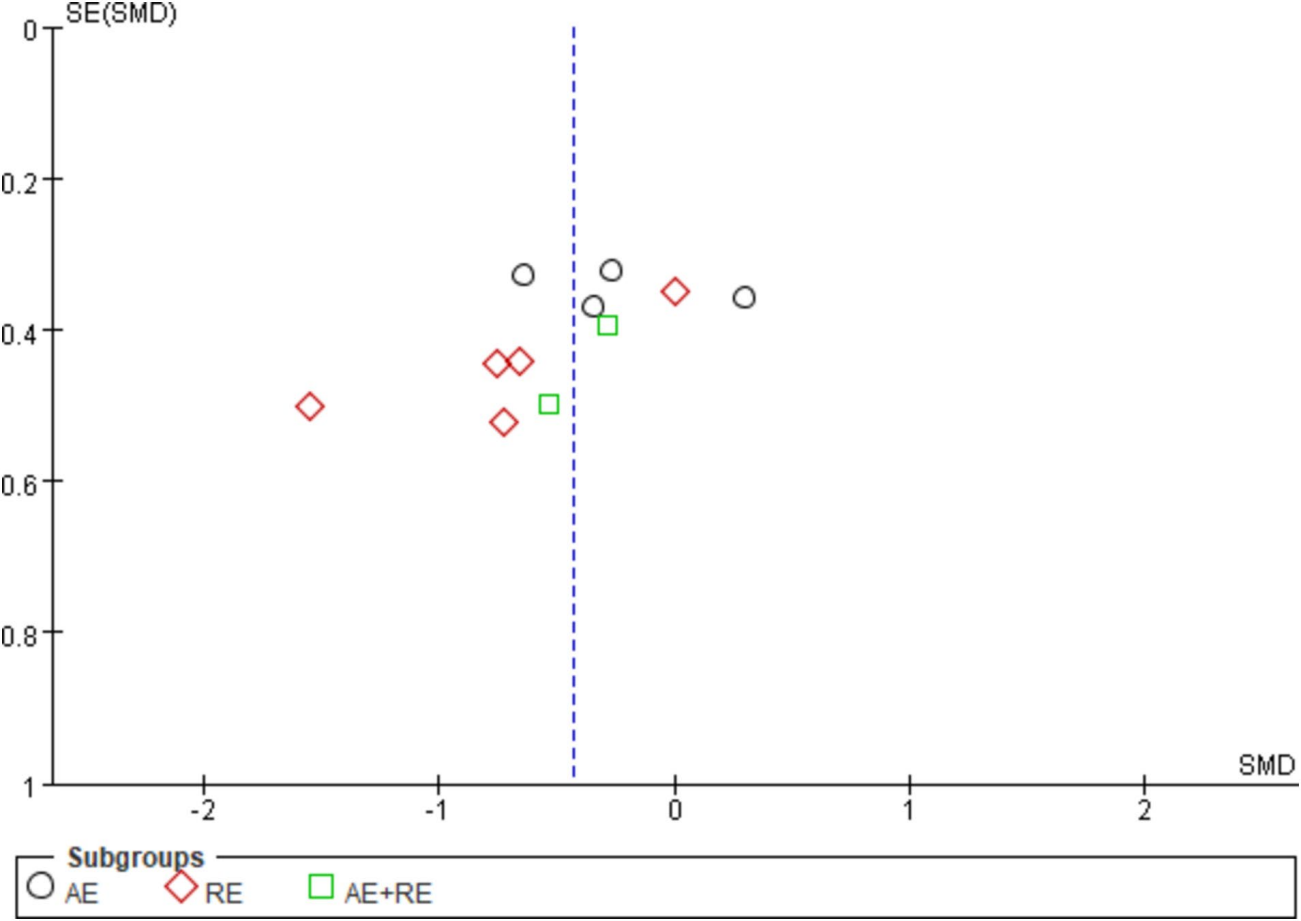
Funnel plot illustrates the effect of exercise on BMI in obese and overweight individuals.

**Figure 9:**
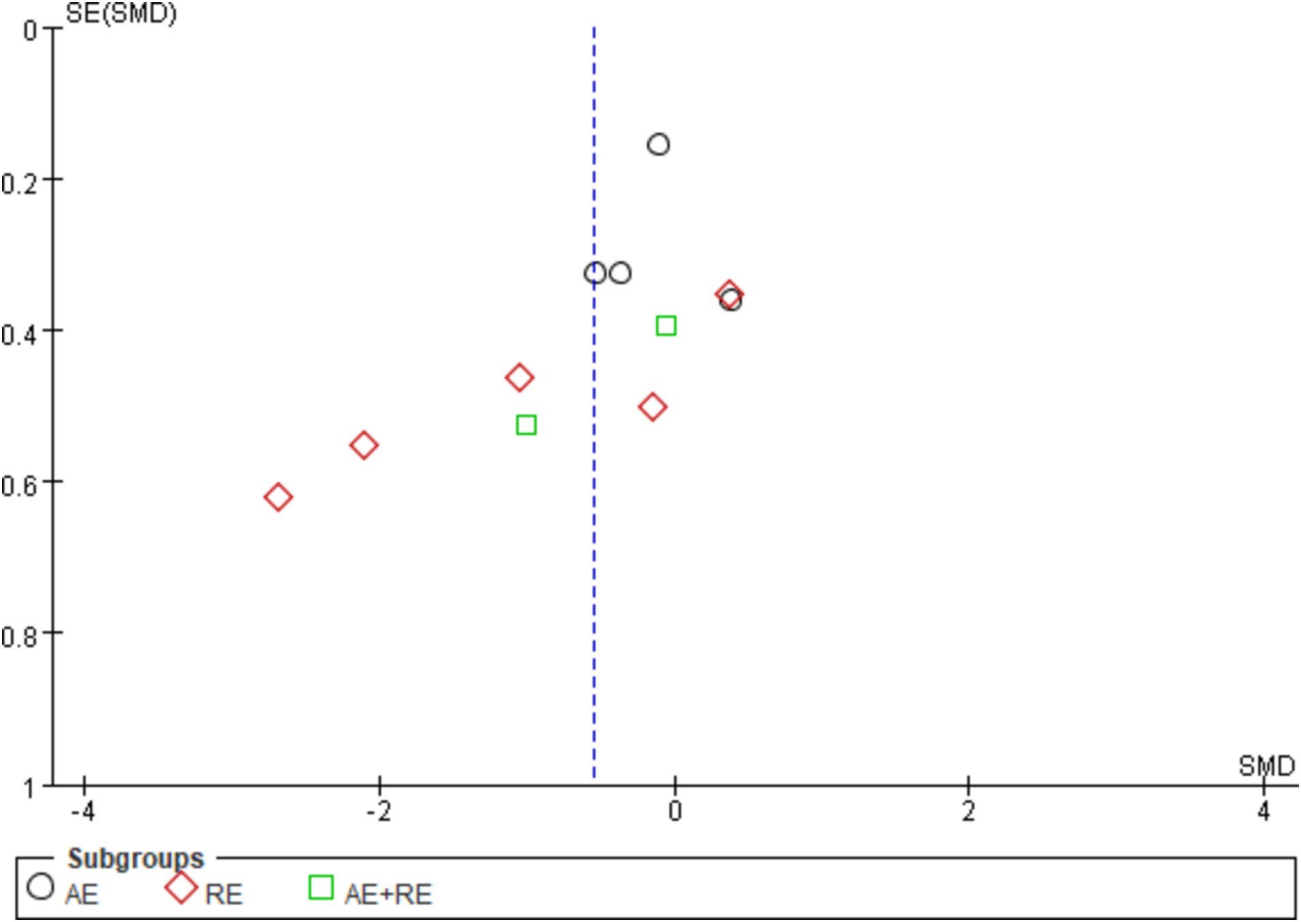
Funnel plot illustrates the effect of exercise on weight in obese and overweight individuals.

The evaluation of publication bias in RevMan software is presented through a funnel plot. In this study, funnel plots were constructed with the standard error (logarithm of SMDs) on the x-axis and the standard error of SMDs (SE(SMDs)) on the y-axis. Funnel plots of the effects of exercise on ghrelin, BMI, and weight are presented below. For ghrelin, the funnel plot shows a slightly asymmetrical distribution (Figure 7). While many of the points are relatively centered, a few studies are scattered at the extremes, suggesting the potential presence of publication bias. After conducting sensitivity analysis, removing a few outlier studies did not significantly alter the overall results, and the heterogeneity remained high, and there was only a slight decrease in heterogeneity after excluding one literature (62). This indicates that the effects of exercise on ghrelin are still statistically significant, and the initial findings are reliable. We hypothesize that this is largely due to differences in how hormones are tested, when exercise is stopped, and the duration of the fasting period. For BMI (Figure 8), the funnel plot shows a fairly symmetrical distribution, with the majority of the studies clustered closely around the central line. There is no obvious sign of publication bias as the points are evenly distributed on both sides of the mean difference. After excluding certain smaller studies to test for bias, the results remained unchanged, indicating that the effect of exercise on BMI is robust and unaffected by potential bias. Weight (Figure 9) shows more variability, with points scattered on both sides of the graph, particularly at the lower end. This asymmetry suggests possible publication bias, especially for studies with larger standard errors. This is mainly due to the fact that some of the eight articles included had the goal of reducing body weight, whereas some did not require a change in the weight of the subjects, all of which contributed to the discrepancies. In conclusion, although some potential publication bias was observed for ghrelin and weight, the sensitivity analyses confirmed the robustness of the overall meta-analysis results for all three indicators. The effect sizes for ghrelin, BMI, and weight remained statistically significant, supporting the validity of the findings.

### 3.6 Meta-regression

The present study conducted a meta-regression analysis to evaluate the effects of BMI changes and weight changes on ghrelin levels. The number of included studies was relatively small, with 11 studies for both BMI and weight, and the heterogeneity was high (BMI: *I*^2^ = 98.59%; weight: *I*^2^ = 98.68%). Additionally, the *R*^2^ values of the models were low, suggesting limited explanatory power. Therefore, caution is needed when interpreting and analyzing the results.

**Figure 10:**
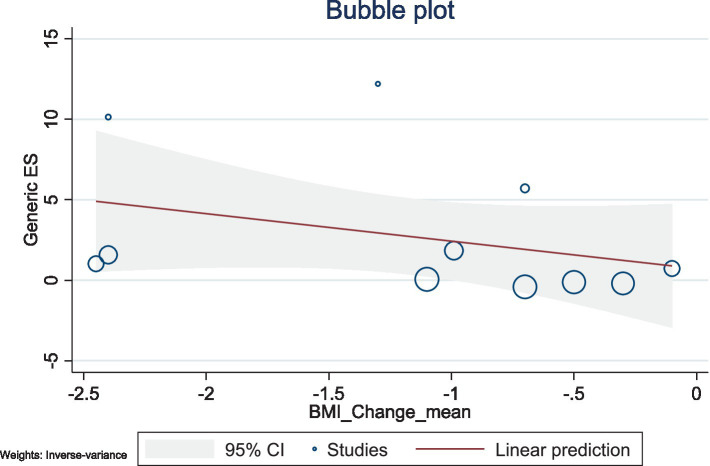
Bubble plot for the meta-regression of BMI changes on ghrelin levels.

**Figure 11:**
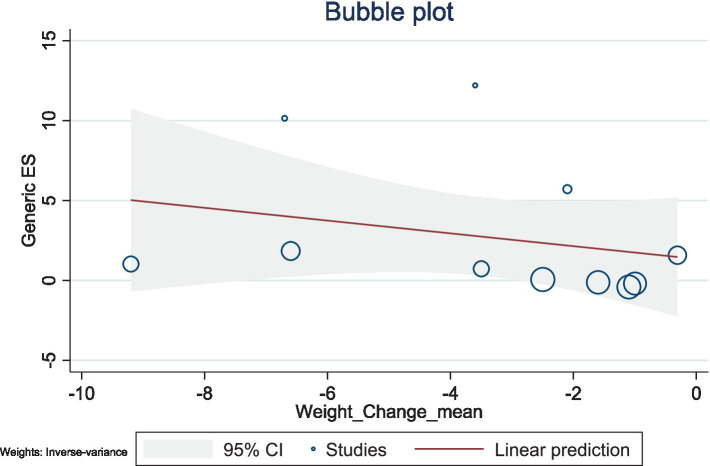
Bubble plot for the meta-regression of weight changes on ghrelin levels.

The results showed a negative association between BMI changes and ghrelin levels (*β* = −1.71, 95% CI: −4.57–1.15, *p* = 0.242), with a low explanatory power (*R*^2^ = 2.27%). Similarly, weight changes also exhibited a negative trend with ghrelin levels (*β* = −0.40, 95% CI: −1.29–0.49, *p* = 0.380), but this association was not significant, with no explanatory power (*R*^2^ = 0.00%). Given the limited number of included studies and the high heterogeneity observed, caution is warranted when interpreting these findings. Future studies should include a larger number of high-quality studies to comprehensively explore the mechanisms underlying changes in ghrelin levels (Figures 10, 11).

## 4 Discussion

This is the first systematic review and meta-analysis to investigate the effects of different types of long-term exercise on ghrelin and to evaluate whether an increase in ghrelin is associated with reductions in BMI and weight. Our findings suggest that long-term exercise significantly impacts ghrelin levels, with RE and AE + RE being the most effective at increasing ghrelin. Our findings indicate that exercise interventions have a statistically significant impact on ghrelin levels in obese or overweight populations, although the overall effect is small, presenting a slight increase in ghrelin levels. Additionally, through the forest plot, we observed that long-term exercise interventions significantly increased ghrelin levels while also significantly reducing BMI and weight. This suggests that the increase in ghrelin levels may be partially associated with the reductions in BMI and weight. We also conducted a meta-regression analysis, which suggested that the increase in ghrelin levels was not related to BMI or weight. However, due to the small number of included studies and high heterogeneity, the reliability of this result is low. Consequently, the explanatory power of our findings is limited, and further research is needed to clarify and validate this relationship.

Our primary finding of an increase in ghrelin supports previous narrative reviews, which suggest that long-term exercise can increase total ghrelin levels (52). However, our results differ from the conclusions of a previous meta-analysis, which claimed that exercise had no effect on ghrelin in obese or overweight individuals (54). The discrepancy may be due to the limited number of studies (only four) included in that meta-analysis and the small amount of weight loss induced by exercise in those studies. By expanding our search to include more literature, we provided stronger evidence that long-term exercise can elevate ghrelin levels. Nevertheless, the high heterogeneity and certain biases indicated by the funnel plot suggest substantial differences between studies. This is mainly due to differences in age, dietary control, food intake, sample collection/processing, timing of blood draws relative to eating and exercise, as well as variations in the assay procedures.

The majority of studies agree that endurance exercise leading to an increase in ghrelin levels is associated with weight reduction in obese and overweight individuals (43, 63–66). Some researchers have noted a strong correlation between changes in ghrelin in human obesity and changes in total weight, waist circumference, and BMI, but not with changes in lean body mass (67). Research has also observed correlations between changes in ghrelin and changes in leptin, adiponectin, insulin, and insulin resistance, which are consistent with the expected direction of weight loss. This supports the role of ghrelin in long-term energy balance. Furthermore, the impact of exercise interventions on fasting ghrelin levels was not altered by baseline adiposity levels (22). Although the exact mechanisms are not yet clear, their effects are likely due to the redistribution of blood flow caused by acute exercise and weight loss resulting from chronic exercise. Some studies have further explained this phenomenon, noting that changes in ghrelin occur when weight loss exceeds 3 kg (22, 68). Therefore, if the weight reduction caused by exercise is minimal, it may not lead to changes in ghrelin levels.

This meta-analysis supports the notion that the rise in ghrelin is correlated with reductions in BMI and weight. From the forest plot, we observed this phenomenon, where long-term exercise interventions increased ghrelin levels while significantly reducing BMI and weight. However, due to the limited number of included studies and high heterogeneity, our meta-regression did not support this viewpoint. The meta-regression results indicated a negative correlation between changes in BMI and weight and ghrelin levels, but these factors were not the primary drivers of changes in ghrelin. A study suggests that, in overweight and obese populations, the overall reduction in body weight is the primary driver of changes in total ghrelin levels. It recommends further research to explore whether exercise can increase ghrelin levels without altering body mass index (BMI) and weight (22). Additionally, our meta-analysis indicates that resistance exercise (RE) or combined resistance and aerobic exercise (RE + AE) is more effective at increasing ghrelin levels than aerobic exercise (AE) alone. First, this may be attributed to changes in BMI and weight. Through subgroup analysis, we found that in the included studies, resistance exercise (RE) and combined aerobic and resistance exercise (AE + RE) were more effective than aerobic exercise (AE) in inducing changes in BMI and weight, which subsequently led to changes in ghrelin levels. Second, this may be associated with mechanisms involving catecholamines as well as differences in exercise modalities and intensities (59). However, the number of studies on RE and AE + RE in our subgroup analysis is relatively small. Future studies with high-quality designs are needed to confirm this finding.

In summary, our conclusions suggest that the compensatory increase in energy intake associated with elevated ghrelin levels following long-term exercise could lead to increased appetite, higher energy intake, and potential weight regain. This systematic review was conducted rigorously according to PRISMA guidelines (55), including dual screening of studies, data extraction, and risk-of-bias assessment. The high heterogeneity and bias suggested by the funnel plot may be due to various factors affecting ghrelin, such as diet, sex, age, blood collection procedures, and individual variability. We found that the included studies varied in the time intervals between exercise and blood sampling procedures for ghrelin (Table 2). If studies did not explicitly define fasting durations or the intervals between exercise and blood sampling, the measured results may reflect acute responses rather than the effects of long-term chronic adaptations (69). We recommend the establishment of standardized methods (22) and adherence to them in the field to facilitate comparisons between studies and reduce heterogeneity. In this review, to assess the correlation between ghrelin and BMI/weight with precision, we included only ghrelin as a hormone. To incorporate more comprehensive data, we used both English and Chinese search terms, but only a few studies met our inclusion criteria (n = 13). Due to the scarcity of high-quality literature, we could not analyze factors such as sex or age on ghrelin. The asymmetry in the funnel plots for BMI and weight was more apparent, mainly because we included fewer studies for BMI and weight, and the intervention durations varied greatly across studies, and it also contains research on children, causing a wide range of fluctuations in BMI and weight, leading to bias in different studies.

There is still a scarcity of high-standard research, and further studies are needed in overweight and obese patients to test the effects of different types of exercise on various appetite hormones, explore the mechanisms by which exercise raises ghrelin in overweight and obese patients, and examine the response of appetite hormones and their impact on energy intake after returning to a normal lifestyle post-exercise intervention to explore and better support weight management and health improvement in overweight and obese populations.

In conclusion, this systematic review and meta-analysis demonstrate that long-term exercise interventions can elevate ghrelin, potentially leading to increased appetite and energy intake after prolonged exercise, resulting in failed weight loss and weight regain. Furthermore, this paper synthesizes existing research findings to show that exercise-induced increases in ghrelin are correlated with reductions in BMI and weight.

## Data Availability

The original contributions presented in the study are included in the article/supplementary material, further inquiries can be directed to the corresponding author/s.

## Footnotes

^1^ https://www.crd.york.ac.uk/prospero/
